# Seasonal piRNA Expression Profile Changes in the Testes of Plateau Zokor (*Eospalax baileyi*)

**DOI:** 10.3390/ani14172620

**Published:** 2024-09-09

**Authors:** Zhiyuan Cai, Baohui Yao, Yuchen Tan, Yongjie Liu, Junhu Su

**Affiliations:** 1 Southwest Survey and Planning Institute of National Forestry and Grassland Administration, Kunming 650031, China; zhiyuancai2022@163.com (Z.C.); sasuke421339218@163.com (Y.T.); 2 College of Grassland Science, Key Laboratory of Grassland Ecosystem, Ministry of Education, Gansu Agricultural University, Lanzhou 730070, China; yaobaohui@nwipb.cas.cn; 3 Key Laboratory of Adaptation and Evolution of Plateau Biota, Northwest Institute of Plateau Biology, Chinese Academy of Sciences, Xining 810008, China

**Keywords:** piRNA–mRNA interaction network, testicular development, seasonal reproduction, spermatogenesis, subterranean rodent

## Abstract

**Simple Summary:**

Seasonal reproduction is a survival strategy employed by many animals, reflecting their adaptation to environmental conditions. This study highlights the differential expression of piRNAs during the reproductive cycle in the testes of plateau zokors (*Eospalax baileyi*) and the associated mRNA enrichment functions and pathways. Our findings demonstrate that piRNAs regulate the PIWI gene family in these high-altitude rodents, thereby influencing testicular development and contributing to seasonal reproduction. The enriched pathways of associated mRNAs regulate and activate testicular development, initiating functions crucial for sperm motility. We believe this study provides valuable insights into the initiation of seasonal reproduction and the underlying regulatory mechanisms in free-living, subterranean rodents.

**Abstract:**

Seasonal reproduction is a mammalian behavior that has developed over an extended evolutionary period and requires animals to respond to external environmental changes to facilitate reproduction. In this study, we investigated the role of PIWI-interacting RNA (piRNA) in the seasonal reproduction of plateau zokors (*Eospalax baileyi*). piRNA expression profiles in plateau zokor testes during both breeding and non-breeding seasons were examined. The piRNAs had a distinctive ping-pong signature and ranged from 27 to 32 nt with a peak at 30 nt. Testicular piRNAs predominantly aligned to specific genomic regions, including repeat and gene regions. Analysis of the piRNA–mRNA interaction network and functional enrichment of differentially expressed piRNAs targeting mRNAs revealed their association with testicular development and spermatogenesis. Significantly, *PIWIL4* is an mRNA gene that interacts with piRNA and exhibits high expression levels within the testes during the non-breeding phase. This study provides a foundation to improve our understanding of piRNA regulatory mechanisms during testicular development and spermatogenesis in seasonally reproducing animals and, specifically, in the plateau zokor.

## 1. Introduction

Non-tropical animals often adopt seasonal reproductive strategies to cope with environmental fluctuations caused by both biological and abiotic factors. This adaptation includes limiting breeding to specific seasons to ensure higher offspring survival rates [1]. Environmental signals such as photoperiod, temperature, food availability, and water play crucial roles in indicating seasonal changes in reproductive status and regulating the rhythm of the endogenous seasonal biological clock [2,3,4]. For many mammals, these factors restrict breeding to spring and summer [5]. During the reproductive season, there is a marked increase in reproductive activity, characterized by gonadal enlargement and elevated baseline sex hormone levels [6,7]. The testes, in particular, regulate paracrine and autocrine molecules produced by the main receptor and germ cells, respectively. Signal molecules and pathways regulating testicular development are essential for the regulation of reproductive development [8,9].

In regulating testicular development, non-coding ribonucleic acids known as PIWI-interacting RNAs (piRNAs) play a critical role in reproductive regulation [10]. During mammalian reproduction, piRNAs are highly expressed in the gonads, contributing significantly to maintaining gamete genome integrity, sex determination, germ cell differentiation, and epigenetic gene regulation [11,12]. Since the identification of small non-coding RNAs, ranging from 25 to 33 nucleotides, in the testicular tissue of *Drosophila melanogaster*, numerous studies have highlighted the important role of piRNAs in reproduction [13,14] The piRNA function and its interaction with genes constitute a mechanism for initiating mammalian reproduction [15]. However, further research is essential to fully understand the complex relationship between piRNA–gene interactions and the reproductive process.

Plateau zokor (*Eospalax baileyi*, Milne-Edwards, 1867), a subterranean rodent, is widely distributed in the alpine meadows of the Qinghai-Tibet Plateau [16]. In recent years, climate change and human activities (overgrazing) have significantly increased the population density of plateau zokors, exacerbating grassland degradation and severely impacting the productivity and ecological functions of these areas [17]. Despite frequent hunting and culling, plateau zokor populations can rapidly rebound, exhibiting a phenomenon of “more and more extinction” [18]. We hypothesize that this phenomenon may be linked to their unique reproductive strategies and high reproductive potential [19]. Plateau zokors exhibit seasonal reproductive changes, primarily reproducing from April to June. Thus, studying their reproductive regulation is necessary to effectively manage their population and apply these mechanisms to control other rodent populations [19]. During non-breeding seasons, plateau zokors experience a significant decrease in testicular weight and testosterone content, with only spermatogonia and supporting cells present in the testes [20]. Recent transcriptomic studies have revealed the substantial effects of differentially expressed genes and microRNAs (DE miRNAs) on the reproductive activity of plateau zokors [21,22]. However, the identification of piRNAs in the testes and their related molecular mechanisms remain understudied.

In summary, we propose that the seasonal reproductive patterns observed in plateau zokors are closely tied to their specialized reproductive strategies, potentially regulated by piRNAs. These piRNAs may play a pivotal role throughout the reproductive cycle by modulating genes and testicular functions associated with reproduction. This regulation likely enables plateau zokors to display seasonal breeding behaviors, allowing them to adapt to the challenging environmental conditions of the Qinghai-Tibet Plateau. In this study, small RNAs obtained through transcriptome technology were utilized to sequence the testicular tissues of male plateau zokors across various reproductive stages in different years. The primary objective of this study is to explore the role of PIWI-interacting RNAs (piRNAs) in regulating reproductive processes across different stages in male plateau zokors. By identifying and analyzing piRNAs in testicular tissues, this research aims to uncover the molecular mechanisms underlying their distinct reproductive strategies and heightened reproductive potential, particularly in response to seasonal environmental changes. The results of this study will enhance our understanding of the regulatory functions of piRNAs in seasonal reproduction, with a specific focus on the plateau zokor.

## 2. Materials and Methods

### 2.1. Animals

This study is situated in the field of reproductive biology, building on the work of Yao et al. [19], with consistent sample information. Live plateau zokors were captured using tube traps (Baoji Ludixincheng Co. Ltd., Xi’an, China) during the breeding season in late April 2020 and the non-breeding season in October 2020 in Tianzhu Tibetan Autonomous County, Gansu Province, China (northeastern Qinghai-Tibet Plateau, alpine meadow-steppe region, 37°19′ N, 102°75′ E). The age structure of wild rodents is typically determined by body or carcass weight, with classifications into sub-adult, adult, and elderly categories [23]. According to Su et al., male plateau zokors are considered adults when their carcass weight exceeds 144 g or their body weight exceeds 180 g [22]. This classification helps to distinguish juveniles from adults in research. Breeding status was assessed by testicular condition, with breeders having relatively larger testes that could be palpated as bulges in the inguinal pockets [24]. In undisturbed sample plots during the breeding season, 74 adult male plateau zokors were captured, with 20 identified as breeders (BSB) and another 20 captured during the non-breeding season (NBS). In total, 20 BSB and 20 NBS individuals were included in the analysis, providing sufficient statistical power to detect the effect of interest. 

Firstly, plateau zokors were euthanized under anesthesia using isoflurane inhalation. Subsequently, their testicles were dissected, weighed, and the data collected were used to assess reproductive status. The intact testes collected were immediately cooled in liquid nitrogen for total RNA extraction and stored at −80 °C for subsequent analysis [15]. This experimental protocol was reviewed and approved by the Animal Ethics Committee of Gansu Agricultural University (approval number GAU-LC-2020-014), and conducted in compliance with the guidelines.

### 2.2. piRNA Extraction, Annotation, and Identification

Three testes close to the mean weight were selected from the BSB (0.118 ± 0.002 g) and NBS (0.037 ± 0.001 g) groups [19]. Raw RNA data were filtered, perfectly mapped reads were retained, and labels were removed. Reads with 1 U or 10 A were considered as candidate piRNAs (Supplementary S1).

### 2.3. Screening of DE piRNAs and Functional Enrichment of Target Genes

piRNAs exhibiting a log2 |fold change| > 1 and a significance level of *p* < 0.05 were identified as showing differential expression. First, we used miRanda and RNAhybrid to predict the target genes messenger ribonucleic acid (mRNAs) of piRNAs. Then, we performed functional enrichment analysis on the identified target genes. The detailed procedures for extracting Gene Ontology (GO) and Kyoto Encyclopedia of Genes and Genomes (KEGG) enrichment analyses, as well as for analyzing differentially expressed messenger RNAs (DE mRNAs), are outlined in Supplementary S1. Subsequently, a targeted association analysis was conducted between DE piRNAs and DE mRNAs, culminating in the construction of a piRNA–mRNA interaction network. Visualization of this network was achieved using Cytoscape software (v. 10.0.1) (Supplementary S1) [24,25].

### 2.4. qPCR Validation

The quantitative polymerase chain reaction (qPCR) was used to verify the expression levels of PIWIL family mRNAs and piRNAs. *Β-ACTIN* and *U6* were selected as reference genes for *PIWIL* and piRNA expression analyses, respectively. The stem-loop method was employed for the design of piRNA primers to enhance detection specificity [14,15]. Reverse transcription stem-loop primers and qPCR amplification primers were subsequently designed and are presented in Table 1 (Supplementary S1).

## 3. Results

### 3.1. Expression of piRNA in the Testes of Plateau Zokors

**Figure 1:**
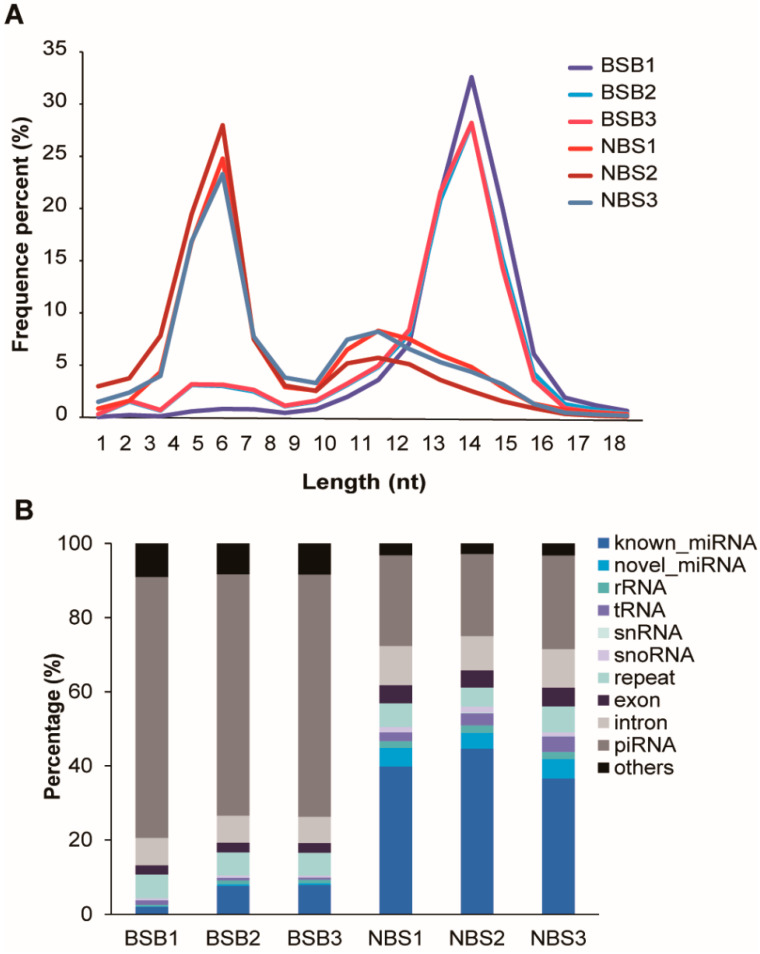
Characterization of piRNAs present in the plateau zokor testis. (**A**) Length distribution of small RNAs. The colors of different lines represent different samples. (**B**) Class of small RNAs with clean reads. Different colors represent differences in the types of small RNAs measured.

Analysis of the small RNA sequencing data indicated two prominent peaks in the mapped reads: one at 21–22 nt, which corresponds to miRNAs, and another at 30 nt, indicative of piRNAs (Figure 1A). Across all sRNA samples, including miRNA, rRNA, tRNA, snRNA, snoRNA, and piRNA, piRNA constituted 22.13–25.24% of the total in the NBS group and 65.11–70.46% of the total in the BSB group (Figure 1B).

**Figure 2:**
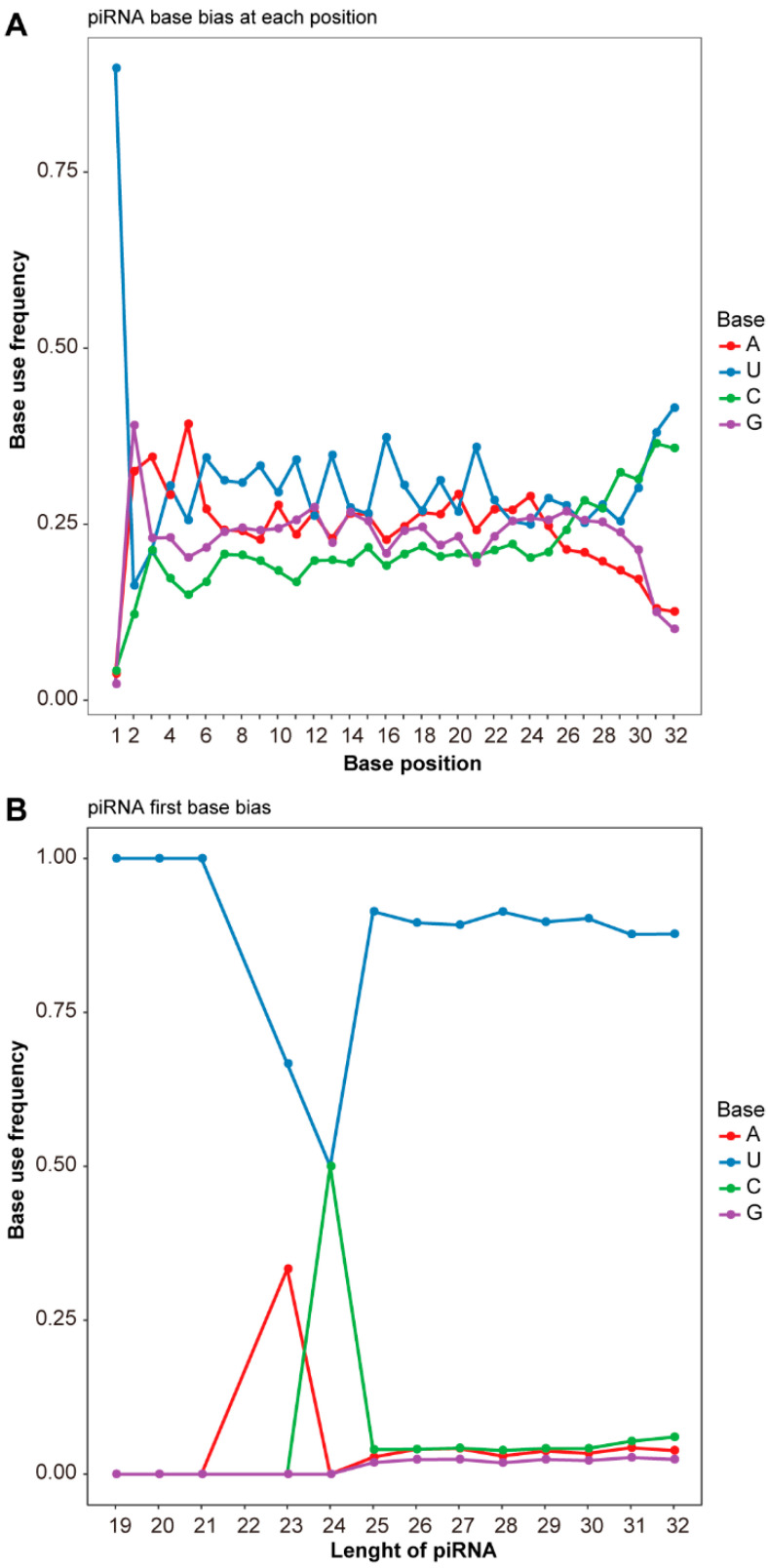
Nucleotide frequencies of piRNAs in the plateau zokor testis. (**A**) Base preference of each site along the 5′-3′ positional nucleotide frequencies of the piRNA sequence. (**B**) Preference of the first base for different piRNA lengths. U, Uridine. A, Adenine. G, Guanine. C, Cytosine.

Statistical analysis conducted using the distribution frequencies of piRNAs in the testes of plateau zokors revealed a pronounced preference for uridine (U) for the first base in the sequence and the 5′ end, whereas the 10th base demonstrated a preference for both U and adenine (A). These findings indicate that piRNAs in the testes of plateau zokors predominantly originate from the primary piRNA pathway (Figure 2A,B).

**Figure 3:**
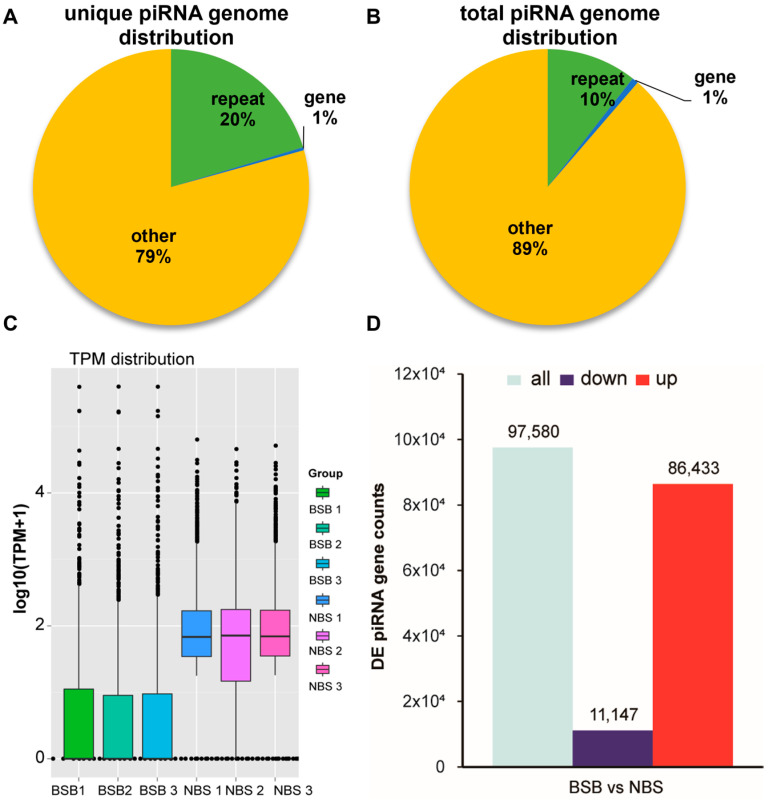
Genomic distribution of plateau zokor testis-derived piRNAs. (**A**,**B**) Genomic distribution of plateau zokor testis-derived piRNAs. (**C**) TPM distribution of samples. (**D**) The number of DE piRNAs. Red represents the number of upregulated differential piRNAs, while dark blue represents the number of downregulated differential piRNAs.

Biological functions of piRNAs vary significantly across different genomic regions. We aligned candidate piRNAs with the plateau zokor genome to identify their primary sources and functional regions. The results showed that piRNAs were primarily aligned with gene domains, repeat regions, and other regions in the plateau zokor testes (Figure 3A,B). This suggests that piRNA plays a crucial role in the reproduction of plateau zokors.

### 3.2. DE piRNAs in the Testes of Plateau Zokors

DE piRNAs during testicular development in plateau zokors were investigated. Expression levels, measured as TPM, across the two piRNA groups (comprising the BSB and NBS data) revealed no aberrant expression in the six samples (Figure 3C). A total of 97,580 DE piRNAs between the BSB and NBS groups were observed, encompassing 86,433 upregulated and 11,147 downregulated piRNAs in the BSB group in comparison with the NBS group (Figure 3D).

### 3.3. Prediction and Enrichment Analysis of Target mRNAs for DE piRNAs

miRanda and RNAhybrid were used to predict their target mRNAs and understand the potential role of DE piRNAs in plateau zokors. The results revealed a total of 486 target DE mRNAs between the BSB and NBS groups.

**Figure 4:**
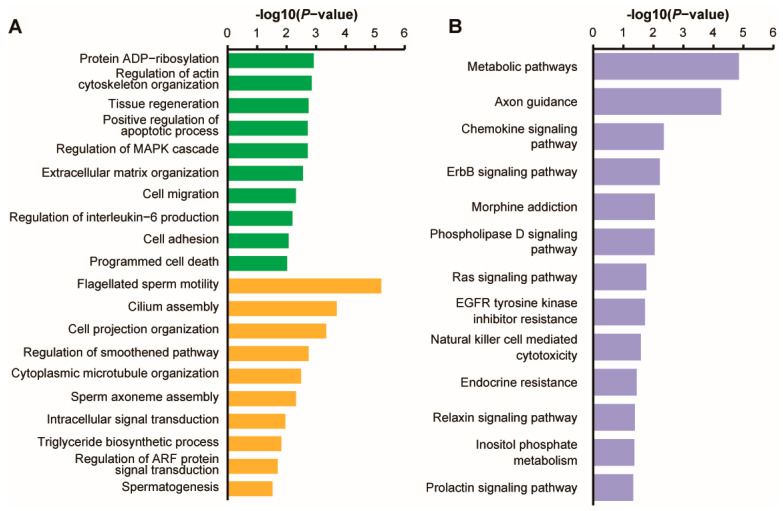
GO and KEGG pathway analyses of the differentially expressed piRNA target genes. (**A**) GO. Green represents the GO terms of downregulated target gene enrichment, while yellow represents the GO terms of upregulated target gene enrichment. (**B**) KEGG.

GO analysis identified the most 10 significantly enriched functions in the downregulated target mRNAs of DE piRNAs between the BSB and NBS groups. These processes included protein ADP-ribosylation, regulation of actin cytoskeleton organization, tissue regeneration, positive regulation of apoptotic processes, regulation of the MAPK cascade, extracellular matrix organization, cell migration, regulation of interleukin-6 production, cell adhesion, and programmed cell death (*p* < 0.05). Similarly, the top 10 enriched biological processes with upregulated target mRNAs included flagellated sperm motility, cilium assembly, cell projection organization, regulation of smoothened signaling pathway, cytoplasmic microtubule organization, sperm axoneme assembly, intracellular signal transduction, triglyceride biosynthetic process, regulation of ARF protein signal transduction, and spermatogenesis (*p* < 0.05; Figure 4A).

In addition, KEGG analysis conducted on the target mRNAs of DE piRNAs identified between the BSB and NBS groups revealed the pathways that were significantly enriched in associated mRNA, such as metabolic pathways, axon guidance, the chemokine signaling pathway, the ErbB signaling pathway, morphine addiction, the phospholipase D signaling pathway, Ras signaling pathway, EGFR tyrosine kinase inhibitor resistance, natural killer cell-mediated cytotoxicity, endocrine resistance, the relaxin signaling pathway, inositol phosphate metabolism, and the prolactin signaling pathway (*p* < 0.05; Figure 4B). Notably, the pathways significantly enriched with target mRNAs did not include any reproductive-related processes. The results confirm that DE piRNAs play a non-redundant role in the development of testicles in plateau zokors.

### 3.4. piRNA–mRNA Interaction Network

**Figure 5:**
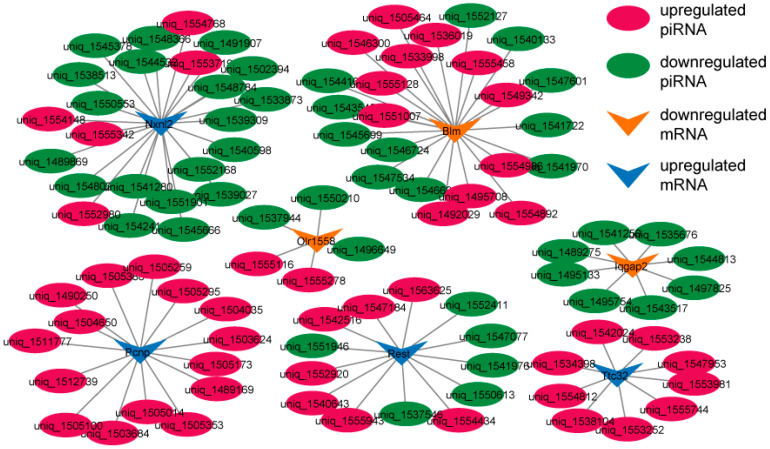
piRNA–mRNA regulatory network in the testes of plateau zokors. The red ellipse represents an upregulated piRNA associated with the target mRNA, while the green ellipse represents a downregulated piRNA associated with the target mRNA.

The piRNA–mRNA regulatory network in the testes of the BSB and NBS groups is shown in Figure 5. The regulatory network delineated the interactions between piRNAs and mRNAs at various stages of testicular development. Such as *NXNL2*, *BLM*, *PCNP*, *TTC32*, *REST*, *IQGAP2*, and *OLR1558* are regulated by multiple differentially expressed piRNAs. The interaction between piRNA and mRNA likely influences the reproductive activity of plateau zokors. This underscores the pivotal role of the piRNA–mRNA association in orchestrating the functional development of animal testes.

### 3.5. Relative mRNA Expression of PIWIL Family Was Measured by qPCR in Plateau Zokor Testes

**Figure 6:**
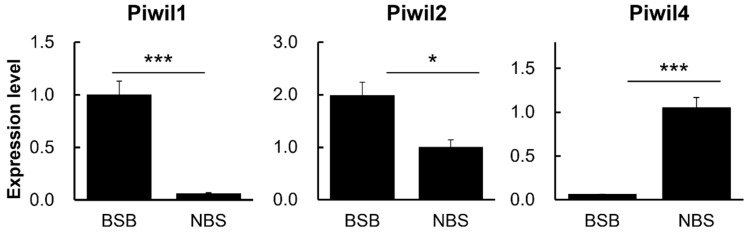
Relative mRNA expression for the PIWIL gene family measured using qPCR in plateau zokor testes. * indicates a significant difference between the two groups, with * representing *p* < 0.05 and *** representing *p* < 0.001.

qPCR analysis of PIWIL family gene expression in testicular tissues from the BSB and NBS groups revealed that, compared to the NBS group, the relative expression levels of *PIWIL1* and *PIWIL2* were significantly higher in the BSB group (*p* < 0.05), while *PIWIL4* expression was significantly lower (*p* < 0.05; Figure 6). These findings suggest a positive correlation between the activation of *PIWIL1* and *PIWIL2* with piRNA regulation during the reproductive process in plateau zokors, whereas *PIWIL4* shows a negative correlation with piRNA regulation.

### 3.6. qPCR

**Figure 7:**
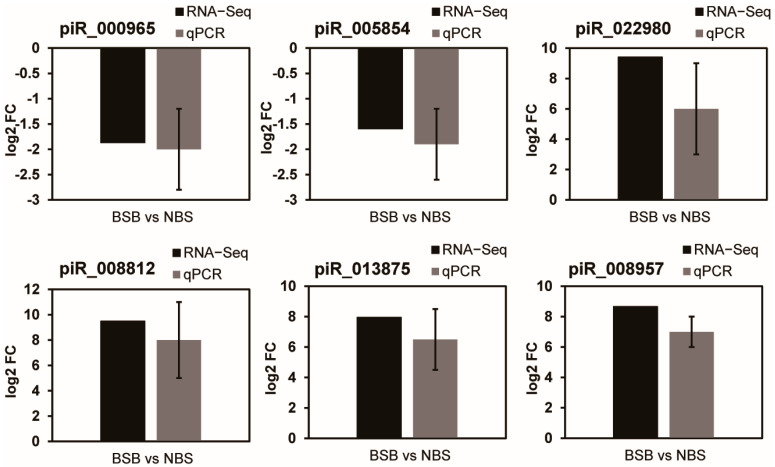
qPCR validation of the piRNA-seq data. The black bars represent the relative expression levels of piRNA in transcriptome sequencing, while the gray bars represent the relative expression levels of piRNA after qPCR validation.

Six known piRNAs (rno-piR000965, rno-piR0008812, rno-piR0008957, rno-piR005854, rno-piR0022980, and rno-piR0013875) were randomly chosen to validate the RNA sequencing data using qPCR. The results demonstrated consistency between the qPCR and RNA sequencing data, indicating the accuracy of RNA sequencing data (Figure 7).

## 4. Discussion

The reproductive capacity of adult animals is largely dependent on the regulation of gene expression regulation during testicular development [11]. Our research supports the hypothesis that PIWI is involved in the regulation of piRNA-mediated reproductive development and gene expression. Specifically, the PIWI gene family during the reproductive development of plateau zokors is regulated by piRNA. We found that piRNA regulation of the RAS, chemokine, phospholipase D, and relaxin signaling pathways activates testicular development, initiating functions related to flagellar sperm motility, cilia components, and sperm axon components. These results indicate that piRNA regulates the PIWI gene family in high-altitude zokors, thereby controlling testicular development and enabling participation in seasonal reproduction. This understanding could inform new strategies for managing rodent populations and mitigating their impact on ecosystems.

### 4.1. DE piRNAs and Differential Gene Association Regulate Testicular Development

Most of the DE piRNAs were upregulated in the BSB group compared to the NBS group, aligning with the anticipated pattern for sexually mature adults. The adult individuals are sexually mature and can participate in reproduction. In this study, piRNAs exhibited a unimodal distribution between 24 and 33 nt in the testes of plateau zokors, with a prominent peak at 30 nt. The length range of piRNAs resembles that observed in the testes of plateau zokors is similar to that observed in the testes of other mammals, such as mice (*Mus musculus*), yaks (*Bos grunniens*), and sheep (*Ovis aries*) [13,14,21]. Mouse testis piRNAs also display a unimodal distribution, with peaks at 26 and 27 nt [24]. Similarly, piRNAs in the testes of rhesus monkeys (*Macaca mulatta*) exhibit a unimodal distribution, with a peak at 29 nt [25]. Previous research has shown that small RNAs in the testes of yak small RNAs showed a bimodal length distribution, with two peaks at 22 nt and >28 nt [26]. This pattern is observed in both sexually immature and mature yaks, highlighting the diverse roles that piRNAs play during different stages of testicular development [26]. Moreover, the estimated length of piRNAs in horse testes ranges from 26 to 32 nt, showing a distinct preference for U at the 5′ end and A at the 10th position [27]. In contrast, the analysis of piRNA base preferences in plateau zokors revealed patterns consistent with those observed in other species. Notably, the first base of all piRNAs shows a strong preference for uridine, indicating their origin from the primary piRNA pathway [27]. The preference for uridine at the first base and adenine at the tenth base aligns with the specificity of endonucleases and the biosynthesis process of the ping-pong cycle [26,28]. Consequently, our analysis confirms the involvement of piRNA in regulating reproductive processes in plateau zokors. 

### 4.2. PiRNAs Participate in the Inhibitory Functions of Transposons in the Testes

piRNAs derived from distinct genomic regions play diverse biological roles [28]. This study revealed that within the genome of plateau zokors, only a limited number of piRNA sets are mapped to repeated sequences and gene regions, while a substantial portion of piRNAs are mapped to unannotated regions [29,30]. The presence of piRNAs in repeat sequence regions of the genome suggests their involvement in transposon silencing [14]. Moreover, the alignment of piRNAs to gene regions implies their role in the regulation of gene expression in the testes [14,15]. However, piRNAs located in other sequence regions of the genome have functions that remain unknown. This study analyzed the comparative regions of piRNAs originating from plateau zokor testes in the genome. The findings revealed that piRNAs were primarily aligned with other regions, repetitive sequence regions, and gene regions in the testes of plateau zokors. In comparison with other species, the proportion of piRNAs aligned with repetitive regions in the testes of plateau zokors (10–20%) is similar to that observed in adult mouse testes (approximately 17%), human testes (approximately 22%), and sheep testes (approximately 15%) [13,15,31]. However, the proportion of piRNAs aligned with gene regions in the testes of plateau zokors (1%) was comparable to that in mouse testes (approximately 1.3%), but lower than that in human testes (approximately 10%) and sheep testes (approximately 27%) [15]. This finding indicates that piRNAs in the testes of plateau zokors may primarily be involved in the inhibition of transposon activity, whereas a smaller fraction is implicated in the regulation of posttranscriptional gene expression. 

### 4.3. Signaling Pathways of DE piRNAs Target mRNAs Involved in Reproduction

In this study, both *PCNP* mRNA and its 15 regulatory piRNAs were simultaneously upregulated, while *TTC32* mRNA and its 9 regulatory piRNAs were also upregulated. In contrast, *LQGAP2* mRNA and its 8 associated piRNAs were downregulated during the breeding season. A single target gene can be regulated by multiple piRNAs, a phenomenon known as multiple regulation [32]. These piRNAs bind to different sites on the same mRNA, collaboratively influencing its expression. This multilayered regulation enhances both the precision and stability of gene expression [28]. Synergistic or competitive interactions among piRNAs likely form complex regulatory networks, finely tuning the expression of the target gene [33].

We observed that the upregulated target mRNAs of DE piRNAs in the BSB group, compared to the NBS group, were significantly enriched in processes related to spermatogenesis. This enrichment included aspects such as flagellar sperm motility, cilia components, and sperm axon components. Furthermore, KEGG enrichment analysis indicated significant enrichment of these DE piRNA-associated mRNAs in pathways associated with testicular development and spermatogenesis, including the RAS, chemokine, phospholipase D, and relaxin signaling pathways. Research underscores the pivotal role of the RAS signaling pathway in modulating cellular processes such as proliferation, survival, and differentiation. After activating the RAF and PI3K pathways, RAS initiates the MEK/ERK and AKT/PKB kinase cascades, which collaboratively influence cell death and proliferation mechanisms [33,34,35]. The overactivation of RAS signaling in mouse testes leads to increased proliferation of spermatogonia in the premeiotic stage, dysregulation of meiotic gene expression, and significant cell apoptosis during meiosis [36]. This is likely because different target genes involved in spermatogenesis interact with extracellular matrix (ECM) receptors and the PI3K-Akt signaling pathway [24]. The results suggest that piRNAs primarily activate the RAS signaling pathway to initiate reproductive processes in plateau zokors. Similarly, in pig testes, candidate piRNAs regulate testicular development through ECM receptor interactions, focal adhesion, and the Wnt/PI3K-Akt signaling pathway [25]. Thus, these findings indicate that piRNAs play a crucial role in activating the reproductive processes of plateau zokors by regulating target mRNAs within reproduction-related pathways.

Research has found that mRNA associated with DE piRNAs in plateau zokors is enriched in the chemokine signaling pathway. Evidence suggests that certain chemokines affect the germ cell population involved in spermatogenesis, thereby affecting the post-meiotic stage. For instance, the signal transduction of C-C motif ligand 12 (*CXCL12*) and its receptor, C-C receptor type 4 (*CXCR4*), regulate SSC activity [37]. Disruption of this axis results in the loss of SSCs in vivo, expression of *CXCR4* in SSCs, and *CXCL12*-supporting cell secretions, which aid in SSC self-renewal. The C-C receptor type 6 (*CCR6*) of C-C motif ligand 20 (*CCL20*) is crucial for the maturation, development, and migration of germ cells. The blockade or absence of *CCR6* leads to a loss of chemotaxis in germ cells toward *CCL20* or supporting cells, and *CCR6* aids germ cells in migrating inward in the seminiferous epithelial cycle [38]. This study demonstrates that DE piRNAs regulate the reproductive process in plateau zokors by modulating supporting cells and spermatogenic epithelial cells.

Phospholipase D (PLD), a member of the signaling enzyme superfamily, predominantly produces secondary messenger phospholipids and plays diverse roles in various cellular pathways. MitoPLD, a member of this family, is anchored on the surface of mitochondria and has dual functions. MitoPLD produces phosphatidic acid that regulates mitochondrial shape by promoting mitochondrial fusion and plays a crucial role in the generation of specialized piRNAs necessary for the development of spermatocytes to inhibit meiosis [39]. Male mice lacking MitoPLD are infertile and exhibit meiotic arrest during spermatogenesis [40]. Spermatogenesis requires piRNA assembly and is impeded by the absence of piRNA components during meiosis. MitoPLD-deficient testes distinctly lack mitochondrial intercellular bonds and do not aggregate or produce piRNAs. MitoPLD was the first confirmed mitochondrial protein involved in the male germ cell differentiation process, thereby introducing a novel molecular mechanism and foundational link to mitochondrial disease [41]. These findings suggest that MitoPLD, an RNase involved in piRNA biogenesis, plays a crucial role in regulating spermatocyte development during reproduction in plateau zokors.

Relaxin, a structural component of the insulin peptide superfamily, comprises RELAXIN-1, RELAXIN-2, INSL3, INSL4, INSL5, and INSL6 and binds to the relaxin family of peptide receptors (RXFP1-4). Relaxin is found in male prostate secretions and positively influences sperm parameters and acrosomal reactions [42]. Relaxin knockout mice exhibit reduced testes and prostate weights, along with delayed sperm maturation. Through its specific receptor, RXFP1, relaxin enhances sperm motility and induces acrosome response and empowerment [43]. The disruption of mouse RXFP1 leads to abnormal sperm development and fertility. RXFP2, a receptor homologous to INSL3, is crucial for the testicular descent of male embryos. The disruption of RXFP2 or INSL3 causes cryptorchidism and infertility. In stromal cells, enhanced *INSL3* expression is a factor involved in germ cell survival [44]. INSL3 traverses the blood–testis barrier, enters the seminiferous chamber and epididymis, and activates specific INSL3 receptors on germ cells. RXFP4, a homologous receptor of INSL5, is highly localized in the sperm tail and less localized in the head region of mouse testes. *INSL5* gene knockout mice exhibit decreased fertility and impaired spermatogenesis [45]. *INSL5* expression in the hypothalamus and testes suggests its role in the hypothalamic–pituitary–gonadal axis and significant effects on reproduction. DE piRNA target mRNAs are implicated in the RAS, chemokine, phospholipase D, and relaxin signaling pathways related to male reproduction, indicating the potential efficacy of these piRNAs. The findings suggest that differentially expressed piRNA target mRNAs govern testicular development during the reproductive process of plateau zokors, impacting sperm function and vitality.

### 4.4. Accumulation of PIWIL4 Obstructs Sperm Meiosis in the NBS Group

To elucidate the molecular regulatory mechanisms of DE piRNAs in the plateau zokors, a piRNA interaction network related to mRNA was constructed. The testis samples from the BSB and NBS groups showed that *REST*, *IQGAP2*, and *BLM* were concurrently regulated by multiple piRNAs. *REST*, expressed in undifferentiated and early differentiated spermatogonia, is believed to play a role in regulating the balance between spermatogonia self-renewal and differentiation [46]. *IQGAP2*, which is highly expressed in adult testes and sperm, contributes to the regulation of calmodulin activity in the testes, thereby mediating acrosomal reactions and sperm fertilization [47]. The BLM protein, which is expressed in premeiotic spermatocytes during spermatogenesis, plays a role in meiotic recombination [48]. This suggests that piRNA and its target genes regulate the development and function of testes and sperm in plateau zokors during the reproductive process. Nevertheless, the mechanism underlying the interactions between these piRNAs and their target genes remains unclear, and further research is required to elucidate the regulatory mechanisms of piRNA–mRNA interactions.

The PIWIL family of proteins forms a PIWIL–piRNA complex, which is crucial for regulating the expression of other genes [49]. Testosterone may also activate piRNAs and PIWI proteins, influencing testicular function in adolescent rats by mediating piRNA expression [50]. Furthermore, the absence of piRNA loci can impair sperm function in males [51]. This effect occurs because these small non-coding RNAs bind specifically to PIWI proteins, forming PIWI–piRNA complexes that regulate numerous protein-coding genes in germ cells, including those involved in cell proliferation, meiosis, and anti-apoptotic processes [52,53]. In small mammals, the subunits of the *PIWIL* gene, including related genes and subunits such as *PIWIL1/2/3/4*, have high expression levels in germ cells [54,55]. This indicates that members of the PIWIL family play a role in regulating mammalian reproductive processes, influencing sperm function and quantity, and modulating the expression of mRNA targets related to sperm. Therefore, we observed significant upregulation in the expression levels of *PIWIL1*, *PIWIL2*, and *PIWIL4* in the BSB group, consistent with previous research in humans [56]. This observation aligns with the known role of *PIWIL4* in DNA methylation, which is highly expressed in the testes of NBS plateau zokors, possibly because of the inhibition of NBS spermatogenesis before meiosis I, which leads to the accumulation of *PIWIL4* [57]. These findings indicate that PIWI piRNAs play a non-redundant regulatory role in the reproduction of plateau zokors.

## 5. Conclusions

To our knowledge, the expression profiles of piRNA in the testes of plateau zokors during different stages of seasonal reproduction were generated for the first time in this study. The piRNAs ranged from 27 to 32 nt, with a peak value at 30 nt, and were primarily aligned with other regions of the genome, repeat regions, and gene regions. Functional enrichment analysis of the DE piRNA target mRNAs revealed their association with testicular development and spermatogenesis. *PIWIL4*, an mRNA gene that interacts with piRNA, plays a pivotal role in regulating testicular activity during the non-reproductive period. These findings offer data support into the regulation of testicular function during seasonal male animal reproduction and sperm production, enhancing our comprehension of how animals manage their reproductive processes throughout the breeding season. Building on these insights, our next step will be to conduct an in-depth analysis of specific piRNAs that show significant differential expression during reproductive and non-reproductive periods. We aim to validate their roles in key pathways associated with testicular function, spermatogenesis, and overall reproductive regulation. By integrating these approaches, we seek to gain a comprehensive understanding of the molecular mechanisms driving seasonal reproduction in plateau zokors, particularly through piRNA-mediated regulation. These findings could have broader implications for understanding seasonal breeding patterns in other species and offer valuable insights for reproductive biology and conservation strategies.

## Figures and Tables

**Table 1:** Details of primer sequences of genes used for qPCR.

Type	piRNAs	Sequences of Stem-Loop Primers and qPCR Amplification Primers
piRNA	piR_000965	RT: GTCGTATCCAGTGCAGGGTCCGAGGTATTCGCACTGGATACGACCCAGAA
		F: ATGTACCGAGGATCGCTTGAGTC
		R: ATCCAGTGCAGGGTCCGAGG
	piR_008812	RT: GTCGTATCCAGTGCAGGGTCCGAGGTATTCGCACTGGATACGACAGAACA
		F: AACCGGTTTGAAGGGAGCATTTG
		R: ATCCAGTGCAGGGTCCGAGG
	piR_008957	RT: GTCGTATCCAGTGCAGGGTCCGAGGTATTCGCACTGGATACGACAGAAGC
		F: ACCGAGGTTAACAGACTGAATTGTG
		R: ATCCAGTGCAGGGTCCGAGG
	piR_005854	RT: GTCGTATCCAGTGCAGGGTCCGAGGTATTCGCACTGGATACGACTAGTGC
		F: AACAGTGGTGCGCTATGCCG
		R: ATCCAGTGCAGGGTCCGAGG
	piR_022980	RT: GTCGTATCCAGTGCAGGGTCCGAGGTATTCGCACTGGATACGACGCTGAG
		F: ATGCGCGCTGATCCTTTTCAAC
		R: ATCCAGTGCAGGGTCCGAGG
	piR_013875	RT: GTCGTATCCAGTGCAGGGTCCGAGGTATTCGCACTGGATACGACGCTGAG
		F: CGTGTAAAATAAGACATTTGTAGCCT
		R: ATCCAGTGCAGGGTCCGAGG
	U6	RT: AACGCTTCACGAATTTGCGT
		F: GCTTCGGCAGCACATATACT
		R: TTCACGAATTTGCGTGTCAT
mRNA	*PIWIL1*	F: GAAGGCAGATGGCTCAGAGGTC
		R: GCTACCTCGTTCCTCTGGATGTC
	*PIWIL2*	F: TCGGAATGACTCTGTGCTGGATG
		R: CGATTCTGTAGGTGCGGTTGTTG
	*PIWIL4*	F: GAAGGCAGATGGCTCAGAGGTC
		R: CCGCTTGGGCTGGCTCAC
	*Β-ACTIN*	F: TTGTGCGTGACATCAAAGAG
		R: ATGCCAGAAGATTCCATACC

## Data Availability

RNA-Seq data were deposited in the China National Center for Bioinformation (PRJCA010603, https://ngdc.cncb.ac.cn/gsa/browse/CRA008119, accessed on 8 February 2022). All other study data were included in this article and/or supporting information.

## Supplementary Materials

The following supporting information can be downloaded at: https://www.mdpi.com/article/10.3390/ani14172620/s1, Additional data on detailed information on the location of the trap and the sequencing and analysis methods of piRNA.
