# Supplementary material for: Seasonal piRNA Expression Profile Changes in the Testes of Plateau Zokor (Eospalax baileyi)
Source: Animals (Basel). 2024 Sep 9;14(17):2620. doi: 10.3390/ani14172620 (PMC11394656; doi:10.3390/ani14172620)
Supplement: Supplementary file 1 [file animals-14-02620-s001.zip › animals-3142855-supplementary.pdf]

## Supplementary Data

# Seasonal piRNA Expression Profile Changes in the Testes of Plateau Zokor (*Eospalax baileyi*)

**Zhiyuan Cai [1,2], Baohui Yao [2,3], Yuchen Tan [1,2], Yongjie Liu [1,*] and Junhu Su [2,*]**

[1] Southwest Survey and Planning Institute of National Forestry and Grassland Administration, Kunming 650031, China; zhiyuancai2022@163.com (Z.C.); sasuke421339218@163.com (Y.T.)

[2] College of Grassland Science, Key Laboratory of Grassland Ecosystem, Ministry of Education, Gansu Agricultural University, Lanzhou 730070, China; yaobaohui@nwipb.cas.cn

[3] Key Laboratory of Adaptation and Evolution of Plateau Biota, Northwest Institute of Plateau Biology, Chinese Academy of Sciences, Xining 810008, China

* Correspondence: liuyongjie203@163.com (Y.L.); sujh@gsau.edu.cn (J.S.)

## MATERIALS AND METHODS

### Site information

Plateau zokors were collected from their natural habitat situated at coordinates 37.114526° N and 103.171024° E in Zhaxi Xiulong Township, Tianzhu Tibetan Autonomous County, Wuwei City, Gansu Province. The collection site represents an alpine meadow grassland characterized by an average elevation of approximately 3100 meters. The region experiences an average annual temperature of 0.1 °C, with precipitation primarily occurring in June, July, and August. The average annual rainfall in this area is 400 mm (Latitude and Longitude details, 2022).

### RNA-seq

Three testis samples each from the BSB, and NBS groups were selected for transcriptome sequencing and sent to Beijing NovoGene Co., Ltd. (Beijing, China) for sequencing. Total RNA was extracted using TRIzol reagent (Invitrogen) according to the manufacturer's instructions.

### Total RNA extraction, Library preparation, and sequencing

Total RNA was isolated from the six testicular tissues with an RNA Nano 6000 Assay Kit (Agilent Technologies, CA, USA) following the manufacturer’s protocols. The Agilent 2100 bioanalyzer and NanoPhotometer spectrophotometer were adopted to assess RNA integrity and concentration.

Subsequently, RNA quality, concentration, and integrity were assessed. A sequencing library was generated using the NEBNext® Multiplex Small RNA Library Prep Set (Illumina®). Total RNA was used as input material for the RNA sample preparations.

Briefly, mRNA was purified from total RNA using poly-T oligo-attached magnetic beads, and cDNA was synthesized using mRNA as a template. Selected cDNA library fragments that were preferentially 370–420 bp in length were purified with the AMPure XP system (Beckman Coulter, Beverly, USA). After PCR amplification, the PCR product was purified with AMPure XP beads, and the library was finally obtained. After construction, the library was initially quantified using a Qubit2.0 fluorometer. qRT-PCR was applied to accurately quantify the effective concentration of the library (higher than 2 nM) to ensure the quality of the library.

After the library was quantified, the different libraries were pooled according to the effective concentrations and the target amounts of data produced and sequenced with the Illumina NovaSeq 6000 machine with 150-bp ends read.

**Transcriptomic data analysis**

The image data measured with the high-throughput sequencer were converted into sequence data (reads) using CASAVA base recognition. Raw data (raw reads) in fastq format were first processed through in-house perl scripts, and clean data (clean reads) were obtained by removing reads that contained adapters, reads containing N bases, and low-quality reads from the raw data. Q20, Q30, and GC content of the clean data were then calculated. All the downstream analyses were based on the clean, high-quality data.

Reference genome (BioProjects: PRJNA254049; *Nannospalax galili*) and gene model annotation files were downloaded from the genome website directly. An index of the reference genome was constructed using Hisat2 (version 2.0.5) and paired-end clean

reads were aligned to the reference genome using Hisat2.

The mapped reads of each sample were assembled with StringTie (version 1.3.3b) in a reference-based approach.

FeatureCounts v1.5.0-p3 was implemented to count the read numbers mapped to each gene, and the FPKM for each gene was calculated based on the length of the gene and read count mapped to the gene.

Differential expression analysis of two conditions/groups (two biological replicates per condition) was performed using the DESeq2 R package (version 1.20.0). Padj $\leq 0.05$ and | log2 (fold-change) | $\geq 1$ were set as the threshold for significant differential expression.

**Construction and sequencing of piRNA library**

Each of the six RNA samples served as input material for the small RNA library. Small RNAs, smaller than 40 nt, were isolated from total RNA using the FlashPAGE fractionator (Ambion). Following the manufacturer's instructions, the NEBNext® Multiplex Small RNA Library Prep Set for Illumina® was employed to generate a sequencing library, incorporating index codes and assigning sequences to each sample. Briefly, the 3' SR adapter of NEB was directly and specifically connected to the 3' end of miRNA, siRNA, and piRNA. Subsequent to the 3' linkage reaction, the SR RT primer hybridized with the excess 3' SR adapter, remaining free post-linkage reaction, thereby converting the single-stranded DNA adapter into a double-stranded DNA molecule. This step is crucial for preventing aptamer dimer formation. Additionally, double-stranded DNA is not a substrate mediated by T4 RNA ligase 1, thereby precluding its

connection to the 5' SR adapter in subsequent linking steps. The 5' adapter connected to the 5' end of miRNAs, siRNA, and piRNA. Subsequently, first-strand cDNA synthesis was performed using M-MuLV reverse transcriptase (RNase H-). PCR amplification utilized the SR Primer and index (X) primers from LongAmp Taq 2X Master Mix and Illumina. The PCR product, ranging from 140-160 bp (small non-coding RNA length plus 3' and 5' aptamers), was purified on an 8% polyacrylamide gel. The purified product was retrieved and dissolved in 8 μL elution buffer. Finally, the quality of cDNA libraries was assessed using DNA high-sensitivity DNA chips on the Agilent Bioanalyzer 2100 system, followed by a 50 bp single-end sequencing on the Illumina Hiseq 2500 platform.

**PiRNA annotation and identification**

Filter the raw RNA data by excluding the following: low-quality readings (readings with Qphred ≤ 5 constituting over 50% of the entire reading length), readings with N content > 10% (N denoting specific information about a base indeterminable at a certain position in the reading sequence), readings incorporating 5' adapter contamination, readings lacking 3' adapter sequence and insertion, and readings containing poly A/T/C/G (poly A/T/C/G potentially arising from sequencing errors). Subsequently, eliminate the 3' adapter sequence and readings exceeding 33 nt from the remaining data to obtain a clean reading. Conduct a comparison between the clean readings and the reference genome (*N. galili*) using Bowtie. Filter out perfectly mapped readings by undertaking the following steps: map to miRbase v21.0 (http://www.mirbase.org/) and perform RepeatMask analysis (http://www.repeatmasker.org/) and Rfam

(http://rfam.sanger.ac.uk/search/) to exclude conserved miRNAs. Screen and filter sequences from protein coding genes, rRNA, tRNA, snRNA, snoRNA, and duplicates. Predict new miRNAs in the remaining readings using Mireap 0.2 and subsequently remove them. Finally, consider the remaining readings with 1U or 10A as candidate piRNAs.

**Screening of differentially expressed piRNAs and functional enrichment of target genes**

Standardize the piRNA expression levels in each sample through Transcripts Per Million (TPM). TPM corrected the length of genes by calculating the reads/gene length aligned to genes to obtain the length corrected expression level reads per kilobase (RPK). Calculate the TPM using the sum of RPKs in the library as the Scale Factor. Employ the DESeq2 program (version 1.4.5) to identify differentially expressed (DE) piRNAs in the testes of plateau zokors. Utilize miRanda and RNAhybrid for predicting the target genes of DE piRNAs, selecting candidate target genes for further analysis from the overlapping results generated by both programs. Target genes for DE piRNAs were identified using DAVID software (https://david.ncifcrf.gov/) for Gene Ontology (GO) and Kyoto Encyclopedia of Genes and Genomes (KEGG) enrichment analysis.

**Quantitative polymerase chain reaction (qPCR) validation**

The technique was repeated three times for each sample, and the relative expression levels of the piRNAs and PIWIL families were calculated using the $2^{-\Delta\Delta Ct}$ method, and one-way analysis of variance (ANOVA) was performed using SPSS 19.0.
